# Visible-Light Driven Photodegradation of Industrial Pollutants Using Nitrogen-Tungsten Co-Doped Nanocrystalline TiO_2_: Spectroscopic Analysis of Degradation Reaction Path

**DOI:** 10.3390/nano12132246

**Published:** 2022-06-30

**Authors:** Sanya Khursheed, Rida Tehreem, Muhammad Awais, Dilshad Hussain, Muhammad Imran Malik, Young Sun Mok, Ghayas Uddin Siddiqui

**Affiliations:** 1 H.E.J. Research Institute of Chemistry, International Center for Chemical and Biological Sciences, University of Karachi, Karachi 75270, Pakistan; sanyakalam@gmail.com (S.K.); ridatehreem595@gmail.com (R.T.); muhammadawais9890@gmail.com (M.A.); dilshadhussain@iccs.edu (D.H.); mimran.malik@iccs.edu (M.I.M.); 2 Department of Chemical Engineering, Jeju National University, Jeju 63243, Korea

**Keywords:** photocatalysis, titania, sol-gel, degradation, co-doped, nanoparticles

## Abstract

The purpose to conduct this research work is to study the effect of photocatalytic degradation by developing cost-effective and eco-friendly nitrogen and tungsten (metal/non-metal) co-doped titania (TiO_2_). The inherent characteristics of synthesized nanoparticles (NPs) were analyzed by Fourier transform infra-red spectroscopy (FT-IR), ultra-violet visible (UV-Vis) spectroscopy, Raman spectroscopy, Field emission scanning electron microscopy (FE-SEM), energy dispersive X-ray spectroscopy (EDX), dynamic light scattering (DLS), X-ray diffraction (XRD) spectrometry, and atomic force microscopy (AFM). Co-doping of metal and non-metal has intensified the photocatalysis trait of TiO_2_ nanoparticles in an aqueous medium. This co-doping of transition metal ions and modification of nitrogen extended the absorption into the visible region subsequently restraining the recombination of electrons/holes pair. The stronger light absorption in the visible region was required for the higher activity of photodegradation of dye under visible light illumination to confine bandgap energy which results in accelerating the rate of photodegradation. After efficient doping, the bandgap of titania reduced to 2.38 eV and caused the photodegradation of malachite green in visible light. The results of photocatalytic degradation were confirmed by using UV/Vis. spectroscopy and high-performance liquid chromatography coupled with a mass spectrophotometer (HPLC-ESI-MS) was used for the analysis of intermediates formed during photocatalytic utility of the work.

## 1. Introduction

The industrial expansion has caused atmospheric, ground, and water pollution, all are toxic and carcinogenic for humans and the environment [1,2,3]. Various types of diseases are being caused due to the increasing level of pollutions. It is also badly affecting climate [4]. There are many methods to reduce these pollutions for example; electrolysis [5], oxidation and anaerobic (reduction) reactions [6], ion exchange mechanism, photocatalysis, bioremediation (use of bacteria, fungi, and algae) [7], filtration/coagulation methods [7,8], adsorption [9], etc. But nowadays, photocatalysis is being widely used for the effective removal of organic contaminants since it not only decomposes the harmful contaminants into harmless byproducts but is also considered a feasible method economically and environmentally. In recent times, titanium dioxide (TiO_2_) nanoparticles are considered to be effective for the photocatalysis of pollutants present in the environment [10,11,12] due to their tremendous properties such as high thermal stability, biocompatibility, cost-effectiveness, and non-toxicity [10,13]. TiO_2_ is a semiconductor with a bandgap of 3.2 eV lying in the ultraviolet (UV) range so this photocatalyst can only be excited by the expensive UV light and can only make use of light with a wavelength below 400 nm and thus UV light is only appropriate since it has a wavelength range of 200–400 nm [14,15]. So, to make the use of visible light and conserve energy various trials were evaluated to shift the absorption of titania from the UV region to the visible region [16,17,18], and thus doping will bring the capacity of TiO_2_ under the visible region of light [13,19]. Fabrication of TiO_2_ photocatalysts with foreign ions is an optimistic approach to extend its response from the UV region to the visible-light region [20,21]. A breakthrough in this regard was made in 2001 [20,22] via the doping of TiO_2_ with nonmetallic anions such as nitrogen [23] which is used as dopant in the adaptation of TiO_2_ photocatalyst [24,25]. Oxygen in the lattice of TiO_2_ is replaced by a nonmetallic anion (i.e., nitrogen atom to generate the energy levels on the top of the valence band of titania, which eventually decreases the bandgap). The bandgap constriction due to the nitrogen atom is not sufficient for the effective use of visible light. So, to enhance the efficiency of visible light absorption a synergic effect is introduced by co-doping which ultimately reduces the electron/hole recombination processes. It is elevated that the rate of photodegradation can be enhanced by doping TiO_2_ with transition metal ions along with nitrogen colloids for both oxidation and reduction reaction [26,27]. Charge separation and photostability will be increased by this collective effect of doping. In this study, Tungsten-nitrogen(N,W) co-doped TiO_2_ nanomaterials were synthesized, characterized, and evaluated as potential candidates since co-doping with W exhibited excellent efficiency for the removal of industrial waste and degradation of organic molecules from water due to the reduction in electron/hole recombination rate [28,29]. It exhibited appropriate behavior for surface reactions including redox and adsorption properties and photo stimulation response under visible light source and the band gap is stressed to 2.3–2.8 eV [1]. The nanoparticles are prepared through the sol-gel process since it is very promising for the fabrication of inorganic and organic-inorganic hybrid nanomaterials. It has several advantages such as it allowing the use of low temperatures during synthesis (<100 °C), better molecular level homogeneity and purity from starting materials and effective control of particle size and shape [30,31,32]. The particles (N, W-TiO_2_) showed organic molecules degradation and decolorization within 30 min of treatment under solar light irradiation. The synthesized (N, W-TiO_2_) nanoparticles are active under solar light, which also makes them enchant for the evolution of self-cleaning coatings.

**Figure 1:**
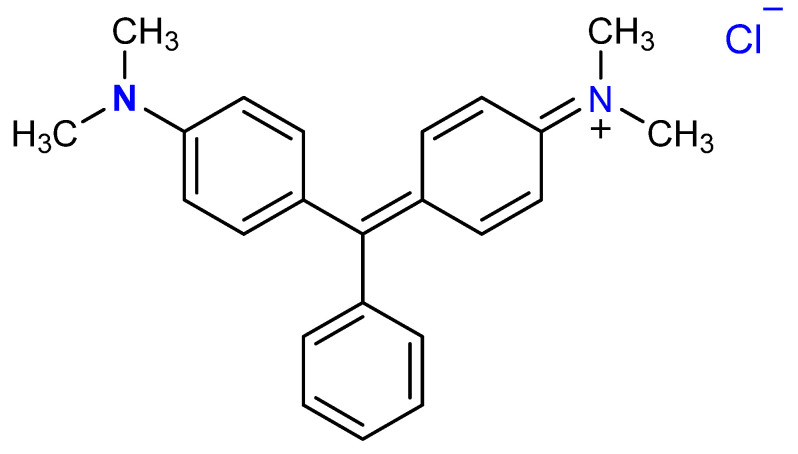
Chemical structure of Malachite Green.

Nowadays, the use of triphenylmethane dye (i.e., Malachite green (MG)) has been extensively increased in many industries due to its easy preparation, cost-effectiveness, and easy accessibility. It is substantially used in many industries. It is not only used in aqua industries but also used as an antibacterial agent, food coloring agent, and an antifungal drug to prevent fungal infections as well as a dye in acrylic, paper, and cotton industries. The structural representation of malachite green dye is shown in Figure 1. Although, the use of this dye has now become highly contested due to its carcinogenic properties and the effects it poses on the immune system and reproductive system of treated fish. The use of this dye has been restricted by US Food and Drug Administration and is preferred for carcinogenic testing [33]. This research emphasis on synthesis and characterization of N, W-TiO_2_ co-doped nanoparticles and their synergic effect on photocatalytic degradation of malachite green dye under visible light.

## 2. Experimental

### 2.1. Materials

Titanium isopropoxide (TTIP) (Sigma Aldrich, Schnelldorf, Germany, CAS: 546-68-9) was used as a source of titanium. 2-Propanol (RCI Labscan), ethylene glycol (Sigma Aldrich, Germany CAS: 107-21-1), and distilled water were used as solvents. Acetic acid (Honeywell, Offenbach am Main, Germany) was used as a catalyst. Tungsten trioxide (WO_3_) (Alfa Aesar CAS: 1314-35-8) was used as a source of tungsten. Urea (CO(NH_2_)_2_) (Honeywell, Fluka) was used as a source of nitrogen. Ammonium acetate (HPLC grade; Sigma Aldrich, Germany), methanol (HPLC grade; Sigma Aldrich, Germany) used for HPLC-ESI-MS analysis. Malachite green (MG) was procured from Sigma Aldrich, Germany, and used without any further purification.

### 2.2. Photocatalysts Preparation

TiO_2_ nanoparticles were prepared by sol-gel route. In a typical procedure, 6.65 mL Titanium isopropoxide (TTIP) was added dropwise in 50 mL propanol (dissolved by magnetic stirring) at room temperature. After 10 min, 2 mL acetic acid was added as a catalyst, and the temperature adjusted to 70 °C. When it reached at 70 °C, 2 mL of ethylene glycol C_2_H_6_O_2_ was added as coolant and stirred at 500 rpm for 30 min. The obtained particles were washed by deionized water, then these composed gel of titania were dried at 100 °C. The attained solids of titania were ground and finally calcined at 500 °C for 2 h.

An impregnation method was used for N-doped and N, W-co-doped TiO_2_ NPs. Exactly, 1 g of the prepared and calcined TiO_2_ powder was suspended in the aqueous solution of urea and its pH was adjusted to 2 by using nitric acid to prepare solution A. Meanwhile, a precised amount of WO_3_ was mixed in 2 mL deionized water with constant stirring then, 2 mL of 0.1 N HCl and 10 mL of 2-propanol were added to this solution to prepare solution B. Now in this solution B; solution A was consequently added dropwise under vigorous stirring. The solution was subsequently stirred for a further 30–60 min and then dried at 100 °C for 12 h. The obtained particles were crushed and calcinated for 3 h at 450 °C.

### 2.3. Study on Point of Zero Charge

For the analysis of the point of zero, 0.1 M NaCl was prepared and its pH was adapted in the range of 2–12 by adding 0.1 M HCl or NaOH. After that, 20 mL of 0.1 M NaCl each was poured in a conical flask, and then 0.1 g of the prepared photocatalyst was added to these solutions. These flasks were kept for 48 h for shaking using a rotary agitator, and afterward, the final pH of the solution was estimated by using a pH meter. Graphs were then plotted for ΔpH vs. pH_initial_.

### 2.4. Photocatalytic Process

**Figure 2:**
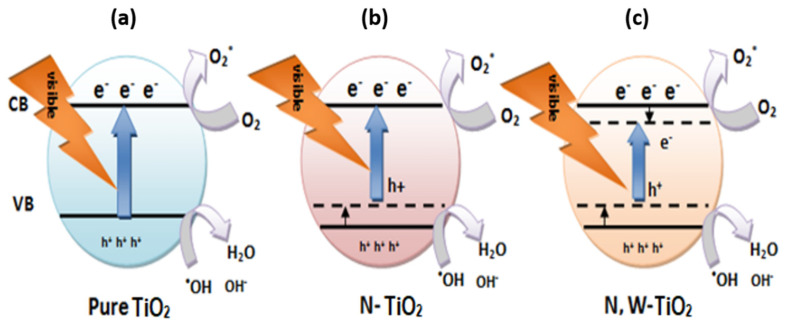
Plausible pathway for the photocatalytic degradation of dye by (**a**) undoped Titania; (**b**) TiO_2_ doped solely with N; (**c**) N, W co-doped TiO_2_.

For the degradation of pollutants without producing harmful intermediates, the most irresistible way is heterogeneous photocatalysis. The first step of the process is the excitation of electrons, the relocation of these electrons from the valance band to the empty conduction band. The activity of photodegradation predominantly depends on the emergence of electron/hole pairs when expose to solar light [34,35]. On the surface of the photocatalyst, an oxidation-reduction reaction takes place where these electron/hole pair migrates and produces reactive oxygen species. Dissolved O_2_ in water trapped the electrons in the conduction band to produce O_2_^−•^ while in the VB h^+^ reacts with surface OH^−^ to form OH^•^. The generated h^+^, O_2_^−•^ and OH^•^ as shown in Figure 2. The generated h^+^, O_2_^−•^ and OH^•^ are considered to be highly active species [36,37,38]. 

For the degradation of malachite green dye solution, photocatalytic activity was evaluated. A photocatalyst was added to the dye solution to prepare a reaction suspension. The pH of this solution was found to be 5.2. To accomplish adsorption-desorption equilibrium this prepared solution was stirred in the dark for 30 min. Later, the solution was irradiated under solar light for different time intervals. The average solar light intensity was about 160 w/m^2^ during mid-summer. The degradation of dye was analyzed by taking 4 mL of the suspension, the catalyst was separated by centrifugation for 10 min at the speed of 15,000 rpm and the concentration of dye in the supernatant solution was estimated using UV-visible spectrum recorded in the wavelength range of 200–800 nm. The rate of degradation was determined from the decrease in absorbance of the dye solution. The removal percentage for the degradation of malachite green was calculated using:

(1) $$\%\;\mathrm{Removal}=\frac{100\times \left(\mathrm{Ci}-\mathrm{Ct}\right)}{\mathrm{Ci}}$$

where C_i_ and C_t_ are the initial and final concentrations at time ‘t’ of dye after UV irradiation.

### 2.5. Separation and Identification of Intermediates

The study of degraded organic intermediates was conducted by HPLC-ESI-MS (Amazon speed procured from Bruker Daltonics). The source used was electrospray ionization with an ion trap analyzer and the detector used was a multi-channel plate (MCP). The solvents used in this experiment are solvent A was 25 mM aqueous ammonium acetate buffer of pH 6.9 while solvent B was methanol, and the flow rate was adjusted at 0.300 mL/min. The gradient profile for LC was Shown in Table 1.

## 3. Results and Discussion

### 3.1. Characterization of NPs

#### 3.1.1. UV-Visible Spectroscopy Analysis

**Figure 3:**
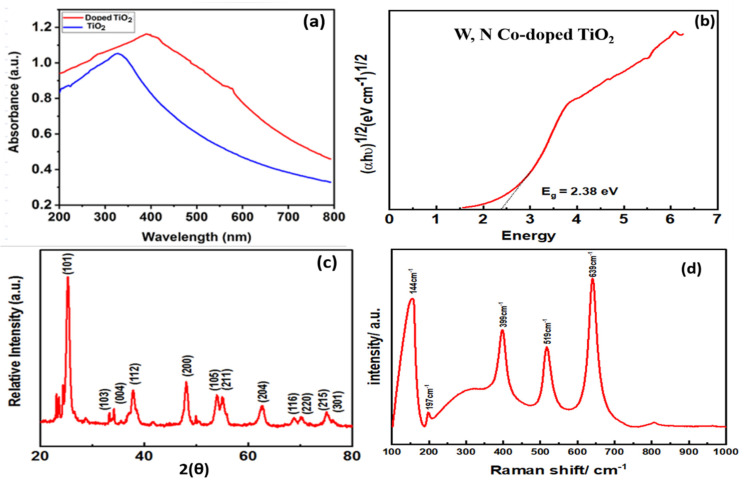
(**a**) UV-vis absorbance spectra of pristine and co-doped TiO_2_ Nanoparticles; (**b**) energy band gap of W, N co-doped titania; (**c**) XRD patterns of co-doped titania; (**d**) Raman scattering studies of co-doped titania.

The UV-Vis analysis confirmed the doping of TiO_2_ NPs by observing a notable redshift in absorbance spectra (334 to ~400 nm) of undoped and doped titania (i.e., from UV-visible region to visible region, as shown in Figure 3a). This doping of TiO_2_ ultimately reduces its bandgap for the photoexcitation (redshift) since new energy levels are formed near the conduction band and valence band which at the same time restrain the recombination of photogenerated electrons/holes pair [2]. Moreover, the reduction in the band gap of TiO_2_ NPs was determined through absorbance spectra by using TAUC plot method for indirect transitions. The obtained indirect band gap value for co-doped titania is 2.38 eV as shown in Figure 3b whereas pristine anatase titania reported 3.2 eV in several studies. This notable reduction in the band gap after the addition of dopants caused the photodegradation of malachite green in visible region.

#### 3.1.2. X-rays Diffraction Analysis

The structural analysis of the NPs was examined by using XRD and data was recorded with 2θ ranging from 10°–80° using Rigaku Benchtop, Cu-kα (λ = 1.5406 Å) radiation. The results of XRD for doped TiO_2_ NPs are shown in Figure 3c confirmed the nanocrystalline structure of doped TiO_2_ NPs due to the sharpness of peaks obtained in spectra. The XRD patterns of co-doped TiO_2_ show diffraction peaks at 25.12°, 33.32°, 34.18°, 37.86°, 48.12°, 53.96°, 54.96°, 62.6°, 69.1°, 70.22°, 75.08°, and 76.2°, these signals showed in XRD pattern are the characteristic signals for anatase phase [39]. The crystal structure of titania [40] and modified titania (i.e., N,W-co-doped TiO_2_ showed similar pattern even the modified titania was calcinated two times at 450 °C). Hence, we can make an affirmation that doping does not affect the anatase phase of titania and its crystal structure.

#### 3.1.3. Raman Scattering Analysis

The crystal phase of co-doped titania was further confirmed by using Raman spectroscopy. The peaks appeared in Raman spectra also indicate the formation of anatase phase of TiO_2_. Six active modes of Raman were observed in the spectra A1g (519 cm^−1^), B1g (399 and 519 cm^−1^) and Eg (144, 197 and 693 cm^−1^) as shown in Figure 3d, all of these modes indicate anatase phase of titania [31]. The slightly broadening and shifting of peak in Raman spectra is due to doping. 

#### 3.1.4. FT-IR Analysis

**Figure 4:**
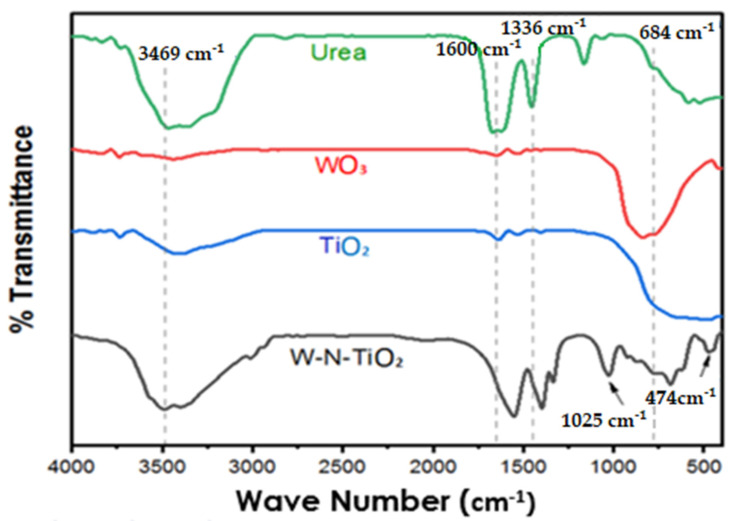
FTIR spectra of urea, WO_3_, TiO_2,_ and tungsten-nitrogen co-doped TiO_2_.

The surface functional groups analysis was carried out by using FTIR, the spectrum confirmed the sample vibrating bonds. The spectra were recoreded from 4000 to 500 cm^−1^, Figure 4 shows the wide absorption peak positioned at 3398–3487 cm^−1^ was attributed to the O-H vibration bond [41,42], the existence of an extensive amount of hydroxyl groups was considered beneficial for the photocatalytic process. The intensive absorption peak appeared at 4734 cm^−1^ belongs to Ti-O [43]. The extremely high transmittance peaks in the region of 1400–1000 cm^−1^ were validated by absorbed molecular oxygen. The peak that appeared in the region of 1550 cm^−1^ was due to the stretching vibrations of the N-H bond which confirms the nitrogen doping [44]. The increase in the intensity of the corresponding TiO_2_ peak confirms the tungsten doping, it increases with the increase in tungsten loading. 

#### 3.1.5. FE-SEM and AFM Analysis

**Figure 5:**
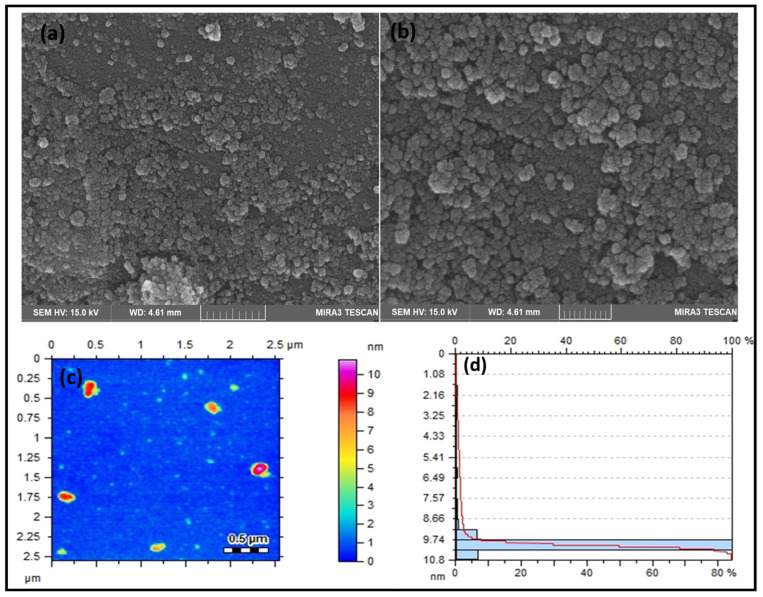
(**a**,**b**) FESEM images of co-doped titania at different scales; (**c**,**d**) AFM analysis of co-doped titania showing size (i.e., ≤10 nm).

FE-SEM images of co-doped titania are shown in Figure 5a,b at different scales. It is evident from these images that there is homogeneity in size and shape of synthesized nanoparticles. The FE-SEM images represent nearly spherical shape of synthesized product (i.e., co-doped titania,), the samples of N, W-TiO_2_ are slightly agglomerated and are more intense. These results demonstrated a vibrant relationship between XRD and SEM results.

The topography and size of the finally synthesized N, W co-doped TiO_2_ nanoparticles were evaluated by AFM (tapping mode) analysis as shown in Figure 5c,d. The particle size distribution shows that large density of nanoparticles have size less than 10 nm.

#### 3.1.6. Energy Dispersive X-ray Analysis (EDX) Analysis

The chemical composition of the photocatalyst was studied through EDX which is given in (Supporting Information Figure S1). The data indicates the presence of nitrogen, tungsten along with titanium and oxygen in the synthesized sample which confirms the doping of titania, the elements are distributed homogenously represented by the elemental mapping of EDX.

#### 3.1.7. Zeta Potential Analysis

**Figure 6:**
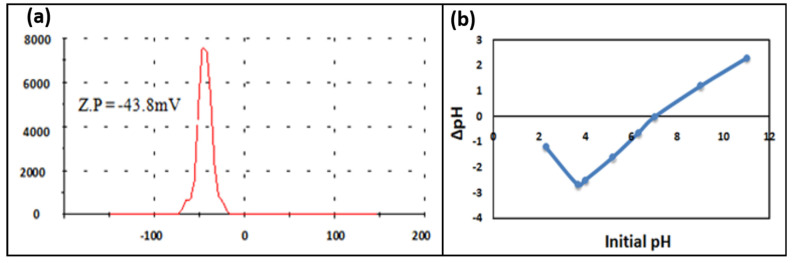
(**a**) Surface charge determination via zeta potential; (**b**) the plot of ΔpH versus pH initial for the determination of pHzpc.

The study of net charges on the surface of NPs is an important aspect since it prevents them from one another to avoid aggregation. The NPs are stable when the net charge is away from zero in either direction. Zeta potential analyzer was used to determine the surface charges on the NPs. The zeta potential of the NPs can be seen in Figure 6a, the net charge on the surface of TiO_2_ was found to be -43.8 eV which shows strong stability of N, W co-doped TiO_2_ NPs.

#### 3.1.8. Point of Zero Charges

When on the surface of particles at certain pH the electrical charge density is zero that point of pH is known as the point of zero charges (pzc) i.e., at this pH it contains as much positively charged as negatively charged surface functions. The surface region below the pzc value will be positively charged (attracting anions); the acidic water donates more proton than OH^-^ groups and therefore the surface of the adsorbent is positively charged. On the contrary, the region above pzc will be negatively charged (attracting cations). Moreover, at zero pzc zeta potential is exhibited by the colloidal system with minimum stability and maximum solubility, maximum viscosity, and other peculiarities.

In this study, the salt addition method was implemented to determine the surface charges for the photocatalyst used in photocatalytic degradation. 0.1 M NaCl was prepared and poured into seven flasks each containing 0.1 g of prepared photocatalyst. Its pH was adjusted in the range of 2–12 by adding 0.1 M HCl or NaOH and then the flasks were shaken for 48 h with a rotary agitator, and the final pH of the solution was determined with a pH meter. The graphs for ΔpH vs. pH_initial_ were then produced. The point of intersection of the curves presented in Figure 6b is the pzc of the adsorbent and it was found to be 7.2. From this data, it was concluded that the surface of NPs will be positively charged when the pH of the solution is less than 7.2 and negatively charged when the pH of the solution is greater than 7.2, so we can say that the malachite green dye was expected to be adsorbed on basic regions by N, W co-doped TiO_2_ NPs.

### 3.2. Dye Degradation Analysis

**Figure 7:**
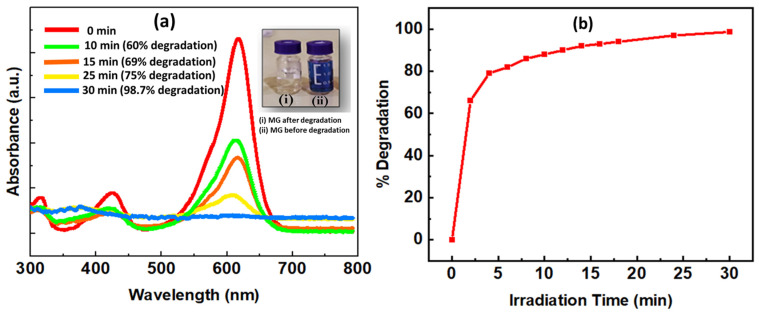
(**a**) UV spectra of N, W co-doped TiO_2_ NPs showing organic molecule degradation within 30 min of treatment under solar light, (**b**) effect of irradiation time on percent degradation of MG.

In the presence of co-doped titania, the dye degradation was studied spectrophotometrically by using a UV-Vis spectrophotometer. The MG dye degradation tests were performed under solar light exposure for 1 h and the changes of absorbance intensity at 618 nm were calculated as shown in Figure 7a. To study the performance of the catalysts, different parameters were applied to optimize their conditions. The particles showed organic molecules degradation and decolorization of dye from the reaction suspension within 30 min of solar exposure.

#### 3.2.1. Potency of the Amount of Photocatalysts on Dye

**Figure 8:**
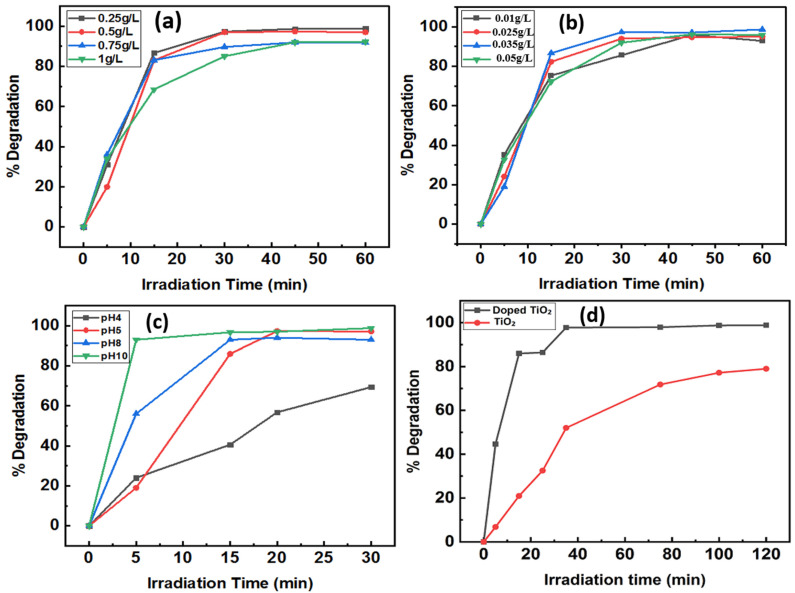
Influence of (**a**) the photocatalysts concentration on the photodegradation mechanism for the decomposition of MG. Experimental conditions: dye concentration (0.25 g·L^−1^), original pH i.e., 5.2, absorbance was recorded at 618 nm, with continuous stirring, irradiation time as 60 min; (**b**) the dye concentration on the rate of photodegradation for the decomposition of MG; (**c**) the pH on the photodegradation mechanism of MG with concentrations of TiO_2_ to be 0.25 g·L^−1^ and MG to be 0.035 g·L^−1^; (**d**) comparison of the rate of photodegradation for the decomposition of MG under TiO_2_ and Co-doped-TiO_2_.

The dependence of the activity of the rate of photodegradation mechanism on the photocatalysts concentration in the MG dye is important to study for its use for practical purposes. Hence, the evaluation in the rate of photodegradation was accomplished by varying different concentrations of adsorbent ranging from 0.25 to 1 g·L^−1^. As predicted, it was observed that the optimum amount of adsorbent required to achieve the highest rate of photodegradation of MG dye was found to be 0.25 g·L^−1^ (Figure 8a). The efficiency was found to be decreased with the increase in the amount of photocatalysts. The photodegradation rate decreased at higher amount of adsorbent may be due to the aggregation of nanoparticles which ultimately decreases the number of surface-active sites. Also, higher amount of adsorbent causes turbidity due to which the rate of degradation decreases as it reduces the penetration of light within the irradiated solution. It causes the light to scattered and deactivate the active photocatalysts by colliding with ground state molecule. 

#### 3.2.2. Concentration of Dye Effect on Decolorization of Dye

The influence of the concentration of dye on the rate of photodegradation could be determined by varying the initial concentration from 0.01 g·L^−1^ to 0.05 g·L^−1^ keeping the catalyst loading constant (i.e., 0.2 g·L^−1^) and the results are shown in Figure 8b. It was found that the degradation efficiency was first decreased then increased with an increase in the concentration of dye and the optimum amount of MG dye for the highest photodegradation rate was found to be 0.035 g·L^−1^. This is because at this concentration the adsorption of the dye molecules is more on the available active sites of photocatalysts and results in donating more electrons to the conduction band of co-doped TiO_2_ nanoparticles. However, at a low concentration of dye, the efficiency was found to be decreased which may be due to the competition of adsorption between the dye and O_2_ molecules which adsorbed on the surface of photocatalysts. Since in photocatalytic reaction the O_2_ molecules which are adsorbed on the surface of photocatalysts are the electron acceptor so the efficiency of photodegradation depends on it. In principle, the amount of dye is proportional to O_2_ molecules. The lesser the number of O_2_ molecules adsorbed on the surface of the dye molecule, the lesser will be the rate of photodegradation. 

#### 3.2.3. Influence of pH on Dye Removal

There is a significant effect of pH on the efficiency and the reaction mechanism of dye photodegradation since the surface charge properties of the photocatalysts are prescribed by the potency of pH. It also gives information about the size of aggregates it forms [45]. The original pH of the malachite green solution was found to be 5.2. The influence of different pH (4, 8, 10) was investigated by adjusting the pH of the solution using 0.1 N HCl and 0.1 N NaOH. From Figure 8c the photocatalytic activity of dye increased from pH 4 to 10 and reached its maximum degradation (i.e., 98% within 5 min of irradiation time using pH 10 solutions). The results showed that the rate of photodegradation of malachite green dye was favorable under basic conditions as the cationic dye causes attraction between the N, W co-doped TiO_2_ NPs and dye molecules. It can easily adsorb on the surface of co-doped TiO_2_ nanoparticles and hence the co-doped-TiO_2_ assisted photodegradation was faster. But in the acidic conditions the solution is accountable for the cleavage of the whole conjugated chromophore structure of the malachite green dye which restricts the dye molecule to adsorb on the surface of nanoparticles and causes repulsion which eventually slowdowns the rate of photodegradation.

#### 3.2.4. Comparison of TiO_2_ and N, W Co-Doped TiO_2_ Degradation Efficiencies

The efficiency test was performed by using commercially available anatase titanium dioxide (P25) compared with the synthesized co-doped TiO_2_ nanoparticles. The experimental results are shown in Figure 8d. The efficiency of anatase titanium dioxide (P25) was 32% in 30 min of irradiation time whereas N, W co-doped TiO_2_ nanoparticles exhibited the best degradation efficiencies of 98% in 30 min exposure to sunlight. This result illustrates that co-doping of semiconductors with metal and non-metal can surpass the TiO_2_ photocatalytic activity under visible light.

### 3.3. Kinetics

To study the adsorption rate of removal, the impact of irradiation time was investigated for the degradation of dye. The percentage removal for the concentration of 0.25 g·L^−1^ with the adsorbent dosage of 0.035 g·L^−1^ at pH 5 is shown in Figure 7b.

To determine the heterogeneous photocatalytic reaction speed and order of reaction between co-doped TiO_2_ based nanoparticles and water-soluble dye, it is important to study the kinetics [3]. To evaluate the kinetics of photodegradation of dyes in the solution, two kinetics models were applied (i.e., pseudo-first order and pseudo-second order) and the following equations were used:

Pseudo-First Order Kinetic Model

(2) $$\log\left(Q_{e}-Q_{t}\right)={\mathrm{logQ}}_{t}-{\;K}_{1}t/2.303$$

Pseudo-Second Order Kinetic Model

t/Q_t_ = 1/h + t/Q_e_ (3)

**Figure 9:**
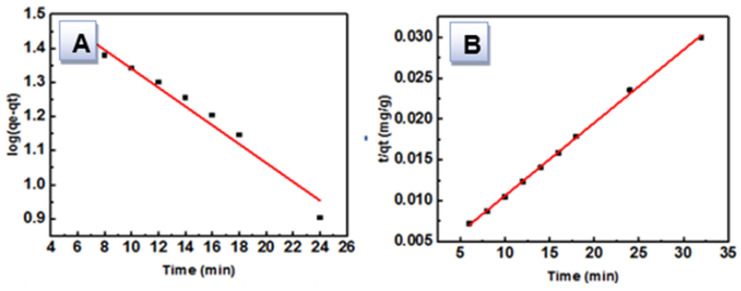
The plots of (**A**) pseudo-first-order kinetic plot and (**B**) pseudo-second-order kinetic plot for the percentage removal for the concentration of 0.25 g·L^−1^ with the adsorbent dosage of 0.035 g·L^−1^ at pH 5 of Malachite green dye.

For the photodegradation of malachite green a slop of log (Q_e_ − Q_t_) versus time (t) graph was fixed for the equilibrium rate constant [k_1_ ($\min^{-1}$)]. The values obtained for pseudo-first order rate coefficient and regression coefficient (R^2^) were −0.0015 (min^−1^) and 0.959 and for pseudo-second order was 4.7 × 10^−4^ and 0.999, respectively, as given in Table 2. The kinetic magnitudes established could be well illustrated by the plot of pseudo-second order shown in Figure 9b. The magnitudes of theoretical and experimental values of equilibrium amounts for the photodegradation of MG dye were 111.19 and 1066 µg g^−1^ respectively, the experimental value was found to be 5% greater than the theoretical one. Also, the R^2^ value of MG for pseudo-second order rate kinetics is 0.999. Therefore, all these conclusions were found to confirm to pseudo-second order kinetic model. Furthermore, the inconsistency in the percentage between the theoretical and experimental values as indicated in Figure 9a for the pseudo-first order was 99%, which is very high. Hence, this observation established the non-fitting of the pseudo-first order kinetic equation. In conclusion, the data for photodegradation of malachite green dye was approved with the pseudo-second order kinetic model.

### 3.4. Reusability Test

**Figure 10:**
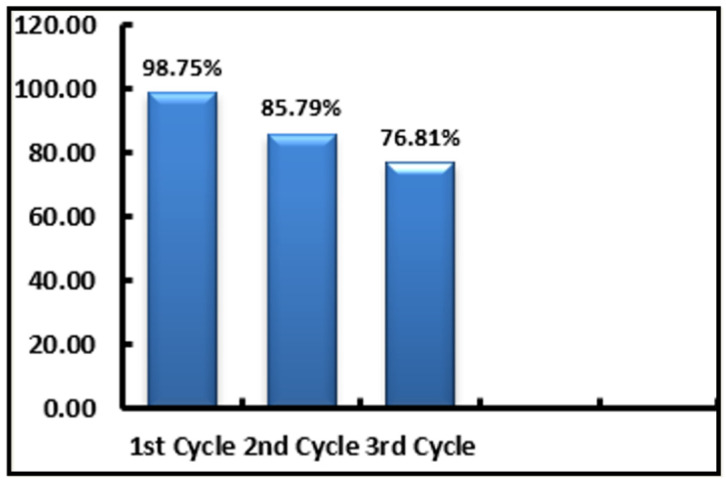
Photodegradation of malachite green dye in cyclic run.

For the practical approach, it is important to study the stability of nanomaterials used during the process of photodegradation. The recyclability was carried out by the repetition of the photodegradation process of MG dye three times using recovered photocatalysts as observed from Figure 10. It is concluded that the efficiency of N, W co-doped TiO_2_ nanoparticles was decreased. Since the active sites on the surface of photocatalysts diminish because of it the equilibrium between adsorption of dye and adsorption of OH^−^ decreases which lowers the efficiency of photodegradation.

### 3.5. Identification of Intermediates Examined by ESI-MS

**Figure 11:**
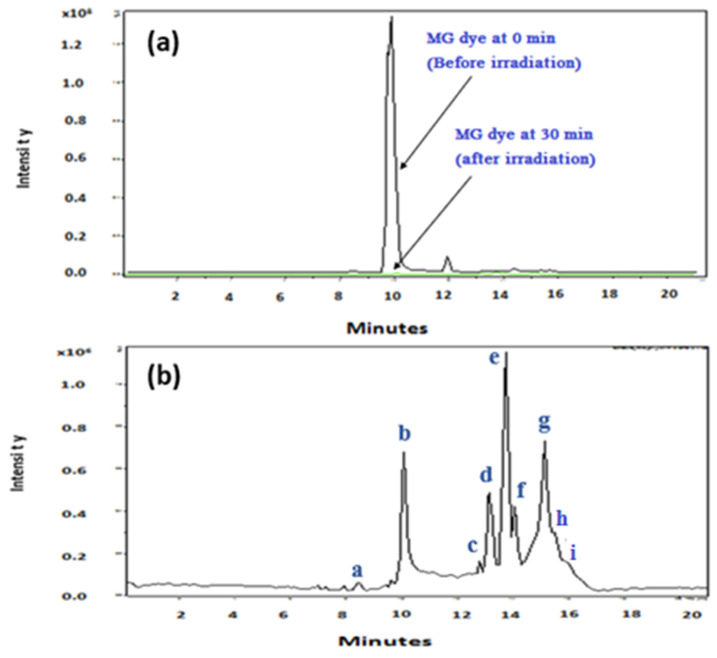
LC-MS chromatogram of (**a**) overlapped chromatogram of MG dye and its degraded products (**b**) the degraded products formed with 30 min of solar irradiation with max of a–h are 618, 620.1, 615.4, 607.9, 598.1, 599.0, 589.6 and 580.2 nm, respectively.

During the process of photodegradation under visible light, different variations took place within the structure of the MG dye. The variations that occurred were examined by HPLC coupled with a photodiode array detector and further confirmation of these intermediates was studied by ESI-MS and LC-MS. Figure 11a shows the chromatogram of malachite green dye before and after the process of degradation and Figure 11b shows the intermediates formed after the degradation of MG dye molecules. The absorption maximum of the spectral bands shifts hypsochromically from 618 nm to 580.2 nm, respectively. 

The relevant mass spectra (Supporting Information Figure S2) confirmed the existence of nine intermediates; the component as identified by HPLC-ESI-MS includes A, *m*/*z* = 329.4 is MG (Bis(p-Dimethylaminophenyl)phenylmethylium) in the liquid chromatogram; B, *m*/*z* = 347 Malachite green carbinol; C, *m*/*z* = 301.3 (p-Methylaminophenyl)(p-methylaminophenyl)phenylmethylium; D, *m*/*z* = 301.3 (p-Dimethylaminophenyl)(p-aminophenyl)phenylmethylium; E, *m*/*z* = 257 an adduct product is formed; F, *m*/*z* = 121 N,N-Dimethylbenzeneamine; G, *m*/*z* = 226 p-Benzoyl-N,N-dimethylaniline; H *m*/*z* = 218 (methylamino-phenyl)-phenyl-methanone; I *m*/*z* = 179 Diphenylmethane. The results of HPLC-ESI-MS are encapsulated in Table 3. Based on the components which are identified by HPLC-ESI-MS, we can suggest a plausible route for the photodegradation of MG dye by the doped TiO_2_ NPs. The dye can be degraded by two ways, in first route MG was hydroxylated to form malachite green carbinol which is further fragmented into two parts; it oxidized and decomposed into p-Benzoyl-*N*,*N*-dimethylaniline and *N*,*N*-Dimethylbenzeneamine. *N*,*N*-Dimethylbenzeneamine mineralizes to form H_2_O AND CO_2_ whereas p-Benzoyl-*N*,*N*-dimethylaniline was decomposed by de-methylation process into the stepwise manner and formed (methylamino-phenyl)-phenyl-methanone and finally converted into Diphenylmethane [4]. MG followed the process of N-demethylation in another route. Due to the presence of dimethylamine groups, the MG dye molecule is most likely to locate near the surface of the NPs which accelerates the process of *N*-demethylation during the early stages. When all the methyl groups are replaced by H then an adduct intermediate is generated through addition reaction at a ratio of 2:1 between *N*-demethylated product and OH radical. The OH radicals generated under solar light emerged directly from the reaction between the holes and surface adsorbed OH^-^ and H_2_O. The presence of hydroxyl species on the MG dye is responsible for further degradation, it favors de-methylation processes. Most of the de-methylation process that occurs on MG dye is by the attack of OH radicals which ultimately is responsible for the photodegradation [5].

**Scheme 1:**
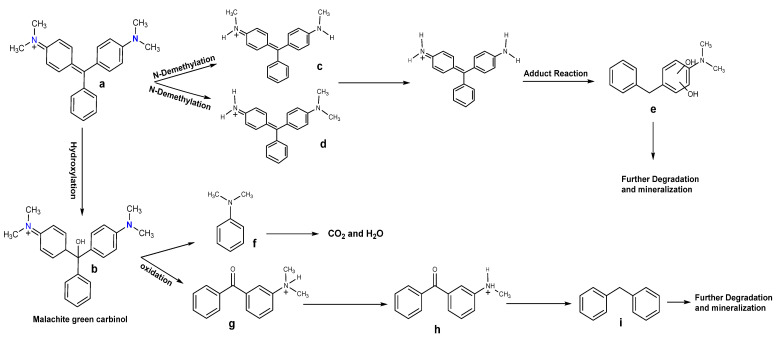
Proposed mechanism of the photocatalytic activity of MG dye under solar irradiation in aqueous Nitrogen and Tungsten doped TiO_2_ dispersions followed by the identification of several intermediates by HPLC-ESI-MS technique.

Following all the above experimental results, an optimistic pathway was proposed for the photocatalytic degradation of MG dye under solar irradiation as illustrated in Scheme 1. In the MG/co-doped TiO_2_ reaction suspension, the positively charged dimethylamine groups adsorbed the molecules of dye. The OH radicals from the surface of co-doped NPs attacked the adsorbed MG via the positively charged dimethylamine groups. The process of demethylation is eventually accelerated by deprotonation which yields a nitrogen-centered radical, which is then attacked by molecular oxygen and aids in the degradation of the dye molecule.

### 3.6. Comparitive Study

Table 4 demonstrates the photocatalytic degradation of MG dye by other workers and with the current study. According to data it is clearly seen that the photocatalytic degradation of MG dye performed by other workers either takes longer time or it requires higher amount of photocatalyst dosage. In some cases the efficiency achieved was lower. In comparision to these studie, under optimized conditions the synthesized N, W TiO_2_ NPs takes shorter time for the degradation of MG dye and no extra treatment was required like temperature, ozonolysis, and pH to achieve high rate of degradation.

## 4. Conclusions

The work presented in this study described details about the synthesis and characterization of modified titania prepared by the sol-gel method for photocatalysis of MG dye. The rate of photodegradation for TiO_2_ NPs co-doped with tungsten and nitrogen is higher than those doped solely with tungsten or nitrogen under solar light since both the metal and non-metal introduced synergic effects in doping of the material. All the material properties including structural and morphological as described through the characterization techniques appeared to be up to the mark. There is successful evaluation of photocatalytic activity and kinetics models for malachite green dye under the radiations of visible light. The highest degradation percentage with good stability after three-run was possessed by N, W co-doped TiO_2_ and it follows the pseudo-second order kinetics with a k_2_ value of 4.7 × 10^−4^ min^−1^. Since this method has proved as adequate, cost-effective, and eco-friendly, it is worthy to be promoted as an approaching photocatalyst technology and the progress towards commercial application.

## Figures and Tables

**Table 1:** Gradient profile for LC to study the degraded organic intermediates for MG dye.

Time	A Phase	B Phase
0	95	5
2	95	5
6	95	5
8	40	60
10	40	60
13	10	90
15	95	5
20	95	5

**Table 2:** Comparison of kinetic parameters associated with pseudo-first order and pseudo-second-order rate equations.

Kinetic Models	Kinetic Parameters	Numerical Values
Pseudo-first-order kinetic model	K_1_ (min^−1^)	−0.00115
	Experimental Q_e_ (µg g^−1^)	1066
	Theoretical Q_e_ (µg g^−1^)	5
	R^2^	0.959
Pseudo-second-order kinetic model	K_2_ (µg g^−1^ min^−1^)	4.7 × 10^−4^
	Experimental Q_e_ (µg g^−1^)	1066
	Theoretical Q_e_ (µg g^−1^)	1119.19
	R^2^	0.999

**Table 3:** Identification of the intermediates formed during the degradation of MG dye by HPLC-ESI-MS.

S. No.	Intermediates	ESI-MS Peaks (*m*/*z*)	Formula
A	Bis(p-dimethylaminophenyl)phenylmethylium	329.4	C_23_H_25_N_2_
B	Malachite green carbinol	347	C_23_H_26_N_2_O
C	(p-Methylaminophenyl)(p-methylaminophenyl)phenylmethylium	301.3	C_21_H_21_N_2_O
D	(p-Dimethylaminophenyl)(p-aminophenyl)phenylmethylium	301.3	C_21_H_21_N_2_O
E	Adduct product	257	D + 2OH
F	*N,N*-Dimethylbenzeneamine	121	C_8_H_1__1_N
G	p-Benzoyl-*N,N*-dimethylaniline	226	C_15_H_15_NO
H	(methylamino-phenyl)-phenyl-methanone	218	C_14_H_13_NO
I	Diphenylmethane	179	(C_6_H_5_)_2_CH_2_

**Table 4:** The activity of nanomaterials for photodegradation of malachite green dye in a previous study.

Photocatalysts	Synthesis Method	Mass of Catalyst (mg)	Efficiency (%)	Time (min)	References
**N, W-TiO_2_**	Sol-gel	25	98.7	30	[This work]
**TiO_2_-Co_2_O_3_**	Sol-gel	120	91.82	60	[37]
**ZnO-La_2_CuO_4_**	Green synthesis (*Strobilanthes crispus*)	2.5	91	120	[46]
**TiO_2_**	Sol-gel	100	80	30	[47]
**Cu**	Green synthesis (Aloe barbadensis Leaf Extracts)	1	70	120	[48]
**Co^2+^-TiO_2_**	Hydrothermal	11.6	82	180	[36]
**TiO_2_**	Synthesized P25 BY Degussa	25	99.9	240	[49]

## Data Availability

Not applicable.

## Supplementary Materials

The following supporting information can be downloaded at: https://www.mdpi.com/article/10.3390/nano12132246/s1. Figure S1. (a) FE-SEM image of co-doped titania selected for EDS analysis, (b) EDS elemental color mapping of finally synthesized co-doped TiO_2_ nanoparticles indicating presence of W, N, Ti and O with respective individual colors, (c) EDS elemental spectrum confirming the doping of W and N in matrix of TiO_2_; Figure S2. The mass spectra of the components as identified by HPLC-ESI-MS. (a) *m*/*z* = 329.4 (Bis(p-dimethylaminophenyl)phenylmethylium); (b) *m*/*z* = 347 Malachite green carbinol; (c) *m*/*z* = 301.3 (p-Methylaminophenyl)(p-methylaminophenyl)phenylmethylium; (d) *m*/*z* = 301.3 (p Dimethylaminophenyl)(p-aminophenyl)phenylmethylium; (e) *m*/*z* = 257 formation of an adduct product; (f) *m*/*z* = 121 N,N-Dimethylbenzeneamine; (g) *m*/*z* = 226 p-Benzoyl-N,N-dimethylaniline; (h) *m*/*z* = 218.1 (methylamino-phenyl)-phenyl-methanone; (i) *m*/*z* = 179.1 Diphenylmethane.
