# Supplementary material for: Visible-Light Driven Photodegradation of Industrial Pollutants Using Nitrogen-Tungsten Co-Doped Nanocrystalline TiO2: Spectroscopic Analysis of Degradation Reaction Path
Source: Nanomaterials (Basel). 2022 Jun 30;12(13):2246. doi: 10.3390/nano12132246 (PMC9267965; doi:10.3390/nano12132246)
Supplement: Supplementary file 1 [file nanomaterials-12-02246-s001.zip › nanomaterials-1771038-supplementary.pdf]

**Supporting Information**

# Visible-Light Driven Photodegradation of Industrial Pollutants Using Nitrogen-Tungsten Co-Doped Nanocrystalline $TiO_2$: Spectroscopic Analysis of Degradation Reaction Path

Sanya Khursheed[1], Rida Tehreem[1], Muhammad Awais[1], Dilshad Hussain[1], Muhammad Imran Malik[1], Young Sun Mok[1,2*], Ghayas Uddin Siddiqui[1,2*]

[1]H.E.J. Research Institute of Chemistry, International Center for Chemical and Biological Sciences, University of Karachi, Karachi-75270, Pakistan

[2]Department of Chemical and Biological Engineering, Jeju National University, Jeju 690-756, Republic of Korea

*Corresponding authors: [1*]smokie@jejunu.ac.kr, [2*]gsiddiqi@jejunu.ac.kr

Tel.: +82-064-754-3682; Fax: +82-64-755-3670

## Characterization Techniques

The morphological studies of N, W $TiO_2$ NPs were conducted by field emission scanning electron microscopy (FE-SEM) (TESCAN, MIRA3, Czech Republic), and elemental doping of W and N were confirmed by energy-dispersive spectroscopy (coupled with FE-SEM) by obtaining EDS spectrum and elemental mapping at 15 kV. The structural, and chemical composition of co-doped $TiO_2$ NPs were analyzed by using XRD equipment (Rigaku Benchtop). UV-visible spectrophotometer (Thermosynthetic evolution 300) was used to investigate the optical properties and extent of photodegradation. Size distribution of co-doped $TiO_2$ NPs were observed by AFM (Agilent technologies 5500). The FTIR analysis is conducted for the collection of the functional groups, present in this synthesized N, W-$TiO_2$ NPs. Zeta potential measurements were made using

Zeta sizer Nano ZS, Malvern Instruments to find out the charge on the surface of NPs. Raman spectra were recorded by a LabRam HR Raman spectrometer with the excitation laser line of 514 nm.

**(a)**

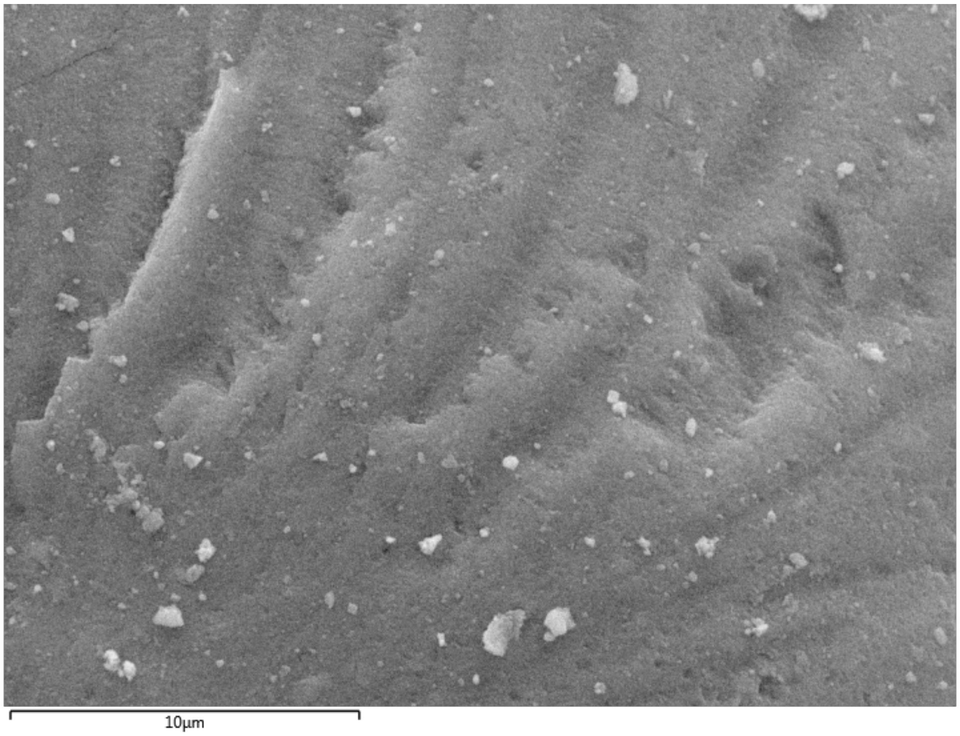

**(b)**

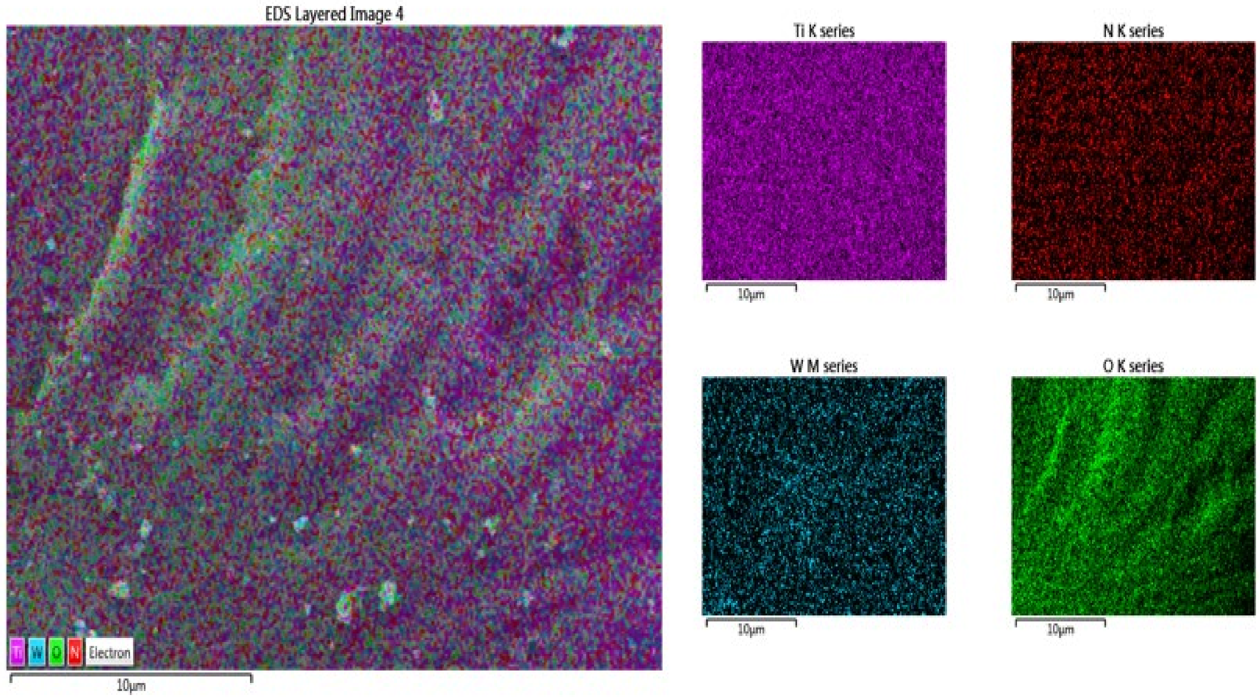
EDS Layered Image 4
Ti
W
O
N
Electron
10μm
Ti K series
10μm
N K series
10μm
W M series
10μm
O K series
10μm

**(c)**

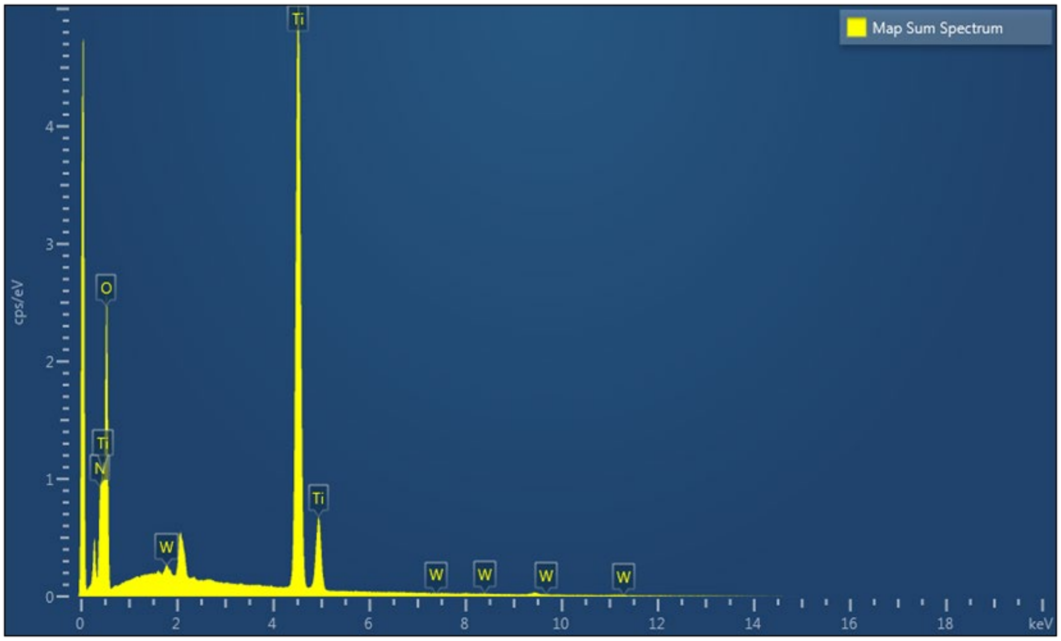
Map Sum Spectrum
cps/eV
keV
Ti
O
Ti
N
W
Ti
W
W
W
W
0
1
2
3
4
0
2
4
6
8
10
12
14
16
18

**Figure S1.** (a) FE-SEM image of co-doped titania selected for EDS analysis, (b) EDS elemental color mapping of finally synthesized co-doped $TiO_2$ nanoparticles indicating presence of W, N, Ti and O with respective individual colors, (c) EDS elemental spectrum confirming the doping of W and N in matrix of $TiO_2$.

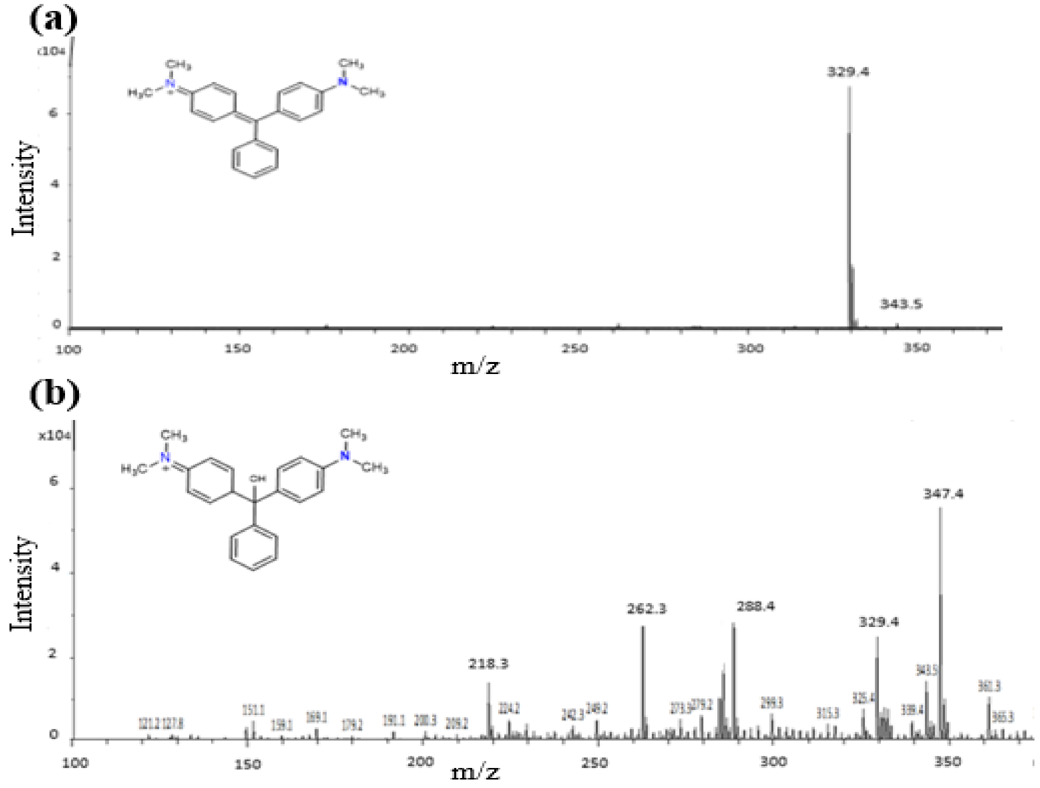

(c)

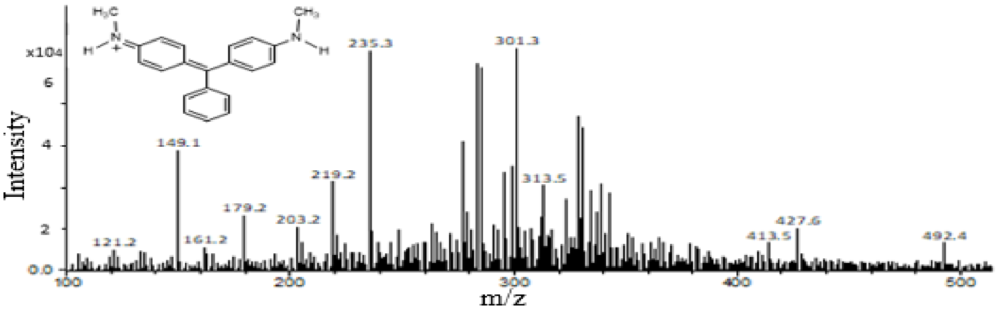

x104
6
4
2
0.0
Intensity
121.2
149.1
161.2
179.2
203.2
219.2
235.3
301.3
313.5
413.5
427.6
492.4
100
200
300
400
500
m/z


(d)

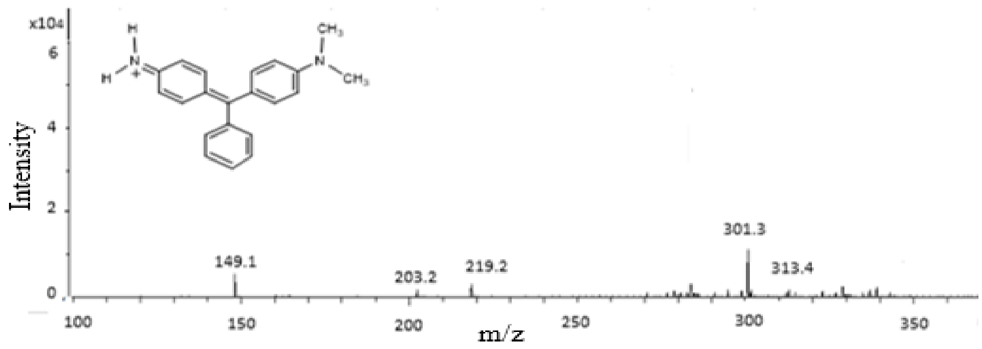

x104
6
4
2
0
Intensity
149.1
203.2
219.2
301.3
313.4
100
150
200
250
300
350
m/z


(e)

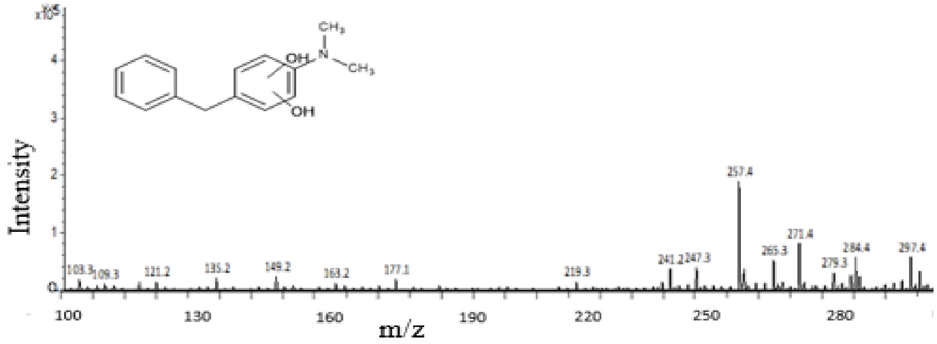

Intensity
103.3
109.3
121.2
135.2
149.2
163.2
177.1
219.3
241.2
247.3
257.4
265.3
271.4
279.3
284.4
297.4
100
130
160
190
220
250
280
m/z

**(f)**

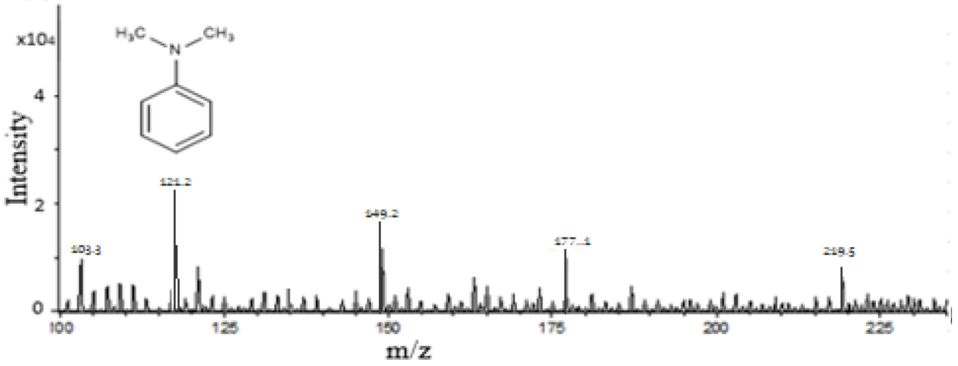

x10⁴
Intensity
m/z
103.3
121.2
149.2
177.4
219.5
0
2
4
100
125
150
175
200
225


**(g)**

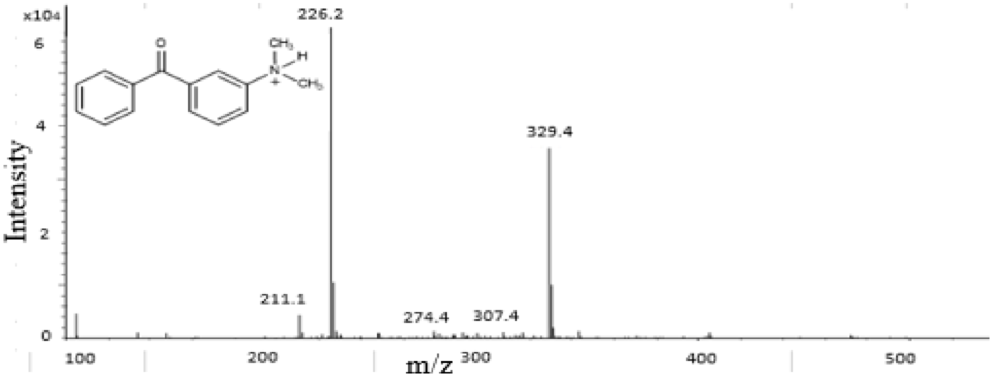

x10⁴
Intensity
m/z
226.2
329.4
211.1
274.4
307.4
0
2
4
6
100
200
300
400
500


**(h)**

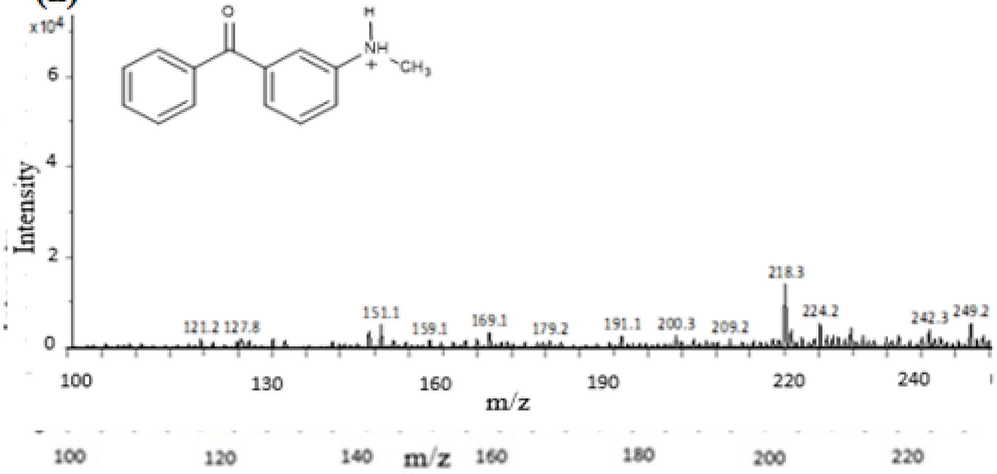

x10⁴
Intensity
m/z
218.3
121.2
127.8
151.1
159.1
169.1
179.2
191.1
200.3
209.2
224.2
242.3
249.2
0
2
4
6
100
130
160
190
220
240

(i)

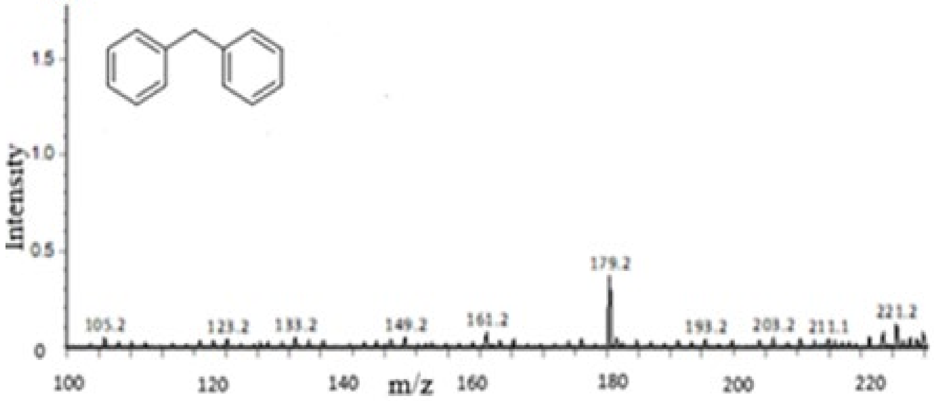


**Figure S2.** The mass spectra of the components as identified by HPLC-ESI-MS. (**a**) m/z=329.4 (Bis(p-dimethylaminophenyl)phenylmethylium); (**b)** m/z=347 Malachite green carbinol; (**c)** m/z=301.3 (p-Methylaminophenyl)(p-methylaminophenyl)phenylmethylium; (**d)** m/z=301.3 (p Dimethylaminophenyl)(p-aminophenyl)phenylmethylium; € m/z=257 formation of an adduct product; **(f)** m/z=121 N,N-Dimethylbenzeneamine; **(g)** m/z=226 p-Benzoyl-N,N-dimethylaniline; (**h)** m/z=218.1 (methylamino-phenyl)-phenyl-methanone; **i** m/z= 179.1 Diphenylmethane.
